# Awareness About the Benefits of Post-bariatric Surgery in Diabetic Patients in Makkah Almukarramah, Saudi Arabia

**DOI:** 10.7759/cureus.48273

**Published:** 2023-11-04

**Authors:** Abdulrahman M Almontashri, Rawan M Almontashri, Khalid Almatrafi, Khalid M Almontashri, Ragad K Aljehani, Mazen S Alshehri, Farraj M Aloqla, Yosra Z Alhindi

**Affiliations:** 1 Pharmacy, Al Mujtama Pharmacy, Makkah, SAU; 2 Medicine and Surgery, Umm Al-Qura University, Makkah, SAU; 3 Community Pharmacy, Aljamaa Pharmacy, Dammam, SAU; 4 Pharmaceutical Care, ​Al-Noor Specialist Hospital, Makkah, SAU; 5 Pharmacy, Umm Al-Qura University, Makkah, SAU; 6 Pharmacology and Toxicology, Faculty of Medicine, Umm Al-Qura University, Makkah, SAU

**Keywords:** awarness, survey, bariatric surgery, type 2 diabetes mellitus, obesity

## Abstract

Aim: To measure awareness about the benefits of post-bariatric surgery, which is a shortening of the gut in order to lose weight in diabetic patients in the Makkah region, Saudi Arabia.

Methods: A cross-sectional survey was conducted among diabetic patients aged 18-65 from February to July 2023. An online questionnaire via Google Forms was distributed and used to assess the participants' awareness of the benefits of post-bariatric surgery for diabetic patients.

Results: Overall, 388 participants (56.40% female, 43.60% male) were surveyed. Most participants (91.5%) showed awareness of gastric sleeve surgery, while a significant proportion (85.8%) recognized obesity as a disease. A majority of participants (80.90%) showed awareness of the association between obesity, diabetes, and high blood pressure. However, only 46.10% of participants showed awareness of eligibility criteria associated with bariatric surgery. The majority recognized the effectiveness of bariatric surgery for weight loss but did not consider it the first choice, emphasizing a preference for non-surgical weight loss strategies.

Conclusion: Participants demonstrated good knowledge about obesity and its implications for health. They also demonstrated good knowledge about bariatric surgery as an effective weight reduction method but expressed a preference for non-surgical methods, which reflects their awareness of the complications of bariatric surgery. However, the results showed a lack of awareness of postoperative indications and lifestyle changes, thus highlighting the need for comprehensive patient education and counseling.

## Introduction

Recently, obesity has become one of the most common disorders found in clinical practice and has significant public health consequences that rise annually in both developed and developing countries [1,2]. Metabolic abnormality in overweight and obese patients contributes to an increased risk of cardiovascular disease (CVD) by increasing plasma, free fatty acids, and triacylglycerols (TAG). Such increases lead to their accumulation in the liver and muscles, which is highly associated with the development of insulin resistance and, thus, to type 2 diabetes mellitus (T2DM), hypertension (HTN), and/or non-alcoholic fatty liver disease, which is strongly associated with CVD and contributes to the development of metabolic syndrome (MS) [3,4]. As a result, obesity can significantly contribute to an increase in morbidity and mortality rates. The extent of the problem of overweight and obesity in Saudi Arabia is overwhelming. Consequently, managing overweight and obesity in Saudi adults has become an important part of the Ministry of Health's broader efforts to establish a comprehensive guiding development program.

There are several ways to measure obesity, but the most common are body mass index (BMI) and waist circumference (WC). The BMI is calculated as weight in kilograms divided by height in meters squared (kg/m^2^) and classified as the following: underweight if the BMI is less than or equal to 18.5 kg/m^2^, normal if the BMI is in the range between 18.5 and 24.9 kg/m^2^, overweight if the BMI is in the range between 25 and 29.9 kg/m^2^, and obesity if the BMI is greater than or equal to 30 kg/m^2^ [5,6]. With increasing weight, the risk of developing diabetes mellitus also increases, especially in individuals with a BMI = 35 kg/m^2^, which is 40-fold higher than that of non-obese individuals [7,8].

General and family medicine practitioners in Saudi Arabia should be concerned with the prevention, early diagnosis, and management of overweight and obesity. The main goal in the management of obesity is to reduce comorbidities, such as CVD, dyslipidemia HTN, and T2DM, while improving quality of life through weight loss. A reduction of 5-10% in body weight will result in several health benefits, including improvement in the control of both blood glucose and blood pressure and increasing HDL while decreasing triglyceride and LDL levels. Management primarily focuses on lifestyle intervention through promoting healthy habits of lifestyle, dietary intervention, and counseling, physical activity, physiological, behavioral, and pharmacological interventions [9-11]. Anti-obesity medications are not considered a lifestyle intervention but may be prescribed as an adjunct for an obese patient who failed to achieve weight loss with lifestyle intervention or for an overweight patient with a BMI of 27 kg/m^2^ with at least one comorbidity [12,13].

Bariatric surgery is considered the most effective measure for the long-term treatment of severe obesity complicated by T2DM. Few clinicians and patients have conversations about these procedures, mainly because of persistent concerns that the short- and long-term risks of surgery outweigh the benefits [13,14]. Although 90% of individuals with type 2 diabetes are obese, a substantially smaller fraction of individuals with obesity develop diabetes [14]. However, obesity is thought to be the strongest risk factor for the development of type 2 diabetes [15]. Recently, there has been a rise in the number of people seeking out bariatric surgery: around 15,000 bariatric procedures are performed each year in Saudi Arabia [16]. Bariatric surgery has demonstrated significant weight loss, improvement in insulin sensitivity, decreased cardiovascular risk, and a decrease in mortality from 40% to 23%. Adults with a BMI of at least 40 and those with a BMI of at least 35 with serious concomitant medical illnesses, such as diabetes, are encouraged to seek bariatric surgery by the US National Institutes of Health [16-17]. To facilitate and improve the management of current complications and metabolic status in overweight and obese individuals, an education program is needed to enhance primary healthcare and increase awareness of obesity and its complications [18-20].

## Materials and methods

The current study was approved by the Institutional Review Board at Umm Al-Qura University, Makkah, Saudi Arabia, with approval number (HAPO-02-K-012-2022-11-1286). This study used a cross-sectional survey study with self-reported responses from February 2023 to July 2023. The inclusion criteria targeted all diabetic patients (ages 18-65) in the Makkah region. The exclusion criteria involve individuals without diabetes mellitus, who are less than 18 years old or older than 65, and who submitted incomplete questionnaires. A self-administered online questionnaire, based on published articles [21,22], via Google Forms was distributed, and information was secured and confidential. The survey's validity and reliability were then tested. To ensure clarity and question comprehensibility, validity was assessed by pilot testing on 25 individuals. The survey covered three sections. The first section included participants’ sociodemographic characteristics. The second section used Yes/No and multiple-choice questions to assess participants’ knowledge of obesity and bariatric surgery indications, benefits, and complications. The third section was specifically for participants who had undergone bariatric surgery to assess its effectiveness as a treatment for diabetes.

According to the General Authority for Statistics in the Kingdom of Saudi Arabia, the total population of the Makkah region is about 6,915,006. The prevalence of diabetes type 2 is estimated at around 35% among the Makkah region population [23]. The required sample size was estimated by using the Raosoft sample size calculator (Raosoft, Inc., Seattle, WA) to be 385 people, with a confidence interval (CI) level of 95% and a margin of error of 5%.

The statistical analysis was done by Statistical Product and Service Solutions (SPSS, version 26) (IBM SPSS Statistics for Windows, Armonk, NY). The categorical sociodemographic data were presented as frequencies and percentages. The chi-square test was used to compare the sociodemographic data and the awareness about bariatric surgery to present frequencies, percentages, and P-values. Logistic regression was constructed to predict awareness about bariatric surgery based on the statistically significant sociodemographic data for each primary outcome. The results of the regression were presented as odds ratios and their respective 95% confidence intervals. A P-value of <0.05 is an indication of statistical significance.

## Results

A total of 388 participants were included in the analysis with 225 participants (58.00%) having type 2 diabetes. The demographic characteristics of the participants are presented in Table 1. The age distribution of the participants indicated that the majority were between 18 to more than 60 years old, with the highest proportion falling within the age groups of 18-23 (22.40%). In terms of gender, the study included a slightly higher number of female participants (56.40%). Most participants were married (50.50%), and most participants were Saudi nationals (88.10%). In terms of residential area, most participants (78.90%) were from the governorates of Makkah. It was observed that most participants had a bachelor's degree (64.90%). Concerning monthly income, the largest proportion of participants fell within the income range of less than 5,000 Saudi Riyals (34.50%). Most of the participants fell in the category of overweight (24.10%).

**Table 1:** Sociodemographic data (n=388)

Parameter	Category	N	%
Age	18-23	87	22.40%
	24-30	77	19.80%
	31-40	71	18.30%
	41-50	68	17.50%
	51-60	60	15.50%
	60-75	25	6.40%
Gender	Male	169	43.60%
	Female	219	56.40%
Marital status	Single	151	38.90%
	Married	196	50.50%
	Divorced	21	5.40%
	Widowed	20	5.20%
Nationality	Saudi	342	88.10%
	Non-Saudi	46	11.90%
Residential area	From the governorates of Makkah	306	78.90%
	Outside the governorates of Makkah	82	21.10%
Educational level	High school education or less	108	27.80%
	Bachelor's	252	64.90%
	High education	28	7.20%
Monthly income	<5,000	134	34.50%
	5,000-10,000	98	25.30%
	10,000-15,000	92	23.70%
	15,000-20,000	38	9.80%
	> 20,000	26	6.70%
BMI	Underweight (18.49 or less)	8	2.10%
	Normal weight (18.5-24.9)	77	20.40%
	Overweight (25-29.9)	91	24.10%
	Class I obesity (30-34.9)	69	18.30%
	Class II obesity (35-39.9)	58	15.40%
	Class III obesity (40 or more)	74	19.60%

Table 2 demonstrates that a total of 225 participants (58.00%) confirmed being diagnosed with type 2 diabetes. Among the diabetic patients, the majority were uncertain about their diabetes medications, with 39.40% responding with "I do not know." Regarding blood sugar level monitoring, 41.80% of the diabetic participants reported checking their blood sugar levels regularly. The duration of type 2 diabetes diagnosis varied among the respondents. Most participants (42.30%) had been diagnosed with diabetes for one year or less. The table shows the respondents' cumulative sugar levels, with the majority falling within the range of <6.5 (34.50%). Regarding awareness of the BMI, slightly over half of the participants (52.60%) confirmed knowing their BMI. A significant proportion of participants (35.60%) were unsure about the range of BMI.

**Table 2:** Data related to diabetes mellitus (DM) (n=388)

Parameter	Category	N	%
Have you been diagnosed with type 2 diabetes?	No	163	42.00%
	Yes	225	58.00%
What diabetes medications do you use?	I do not know	153	39.40%
	Treatment with metformin, a "sugar regulator"	108	27.80%
	Oral glucose-lowering medications	133	34.30%
	Insulin therapy "needle"	63	16.20%
Do you check your blood sugar level regularly?	No	125	32.20%
	Sometimes	101	26.00%
	Yes	162	41.80%
How long were you diagnosed with type 2 diabetes? "in years"	1 year or less	164	42.30%
	2-4 years	88	22.70%
	5-9 years	76	19.60%
	10 years or more	60	15.50%
What is your cumulative sugar level?	<6.5	134	34.50%
	6.5-7.5	76	19.60%
	7.5-8.5	84	21.60%
	8.5-9.5	52	13.40%
	9.5-10.5	23	5.90%
	>10.5	19	4.90%
Do you know the Body Mass Index?	No	184	47.40%
	Yes	204	52.60%
What is the body mass index numerical range in which a person is obese?	I do not know	138	35.60%
	<18.5	23	5.90%
	18.5-24.9	32	8.20%
	25-29.9	84	21.60%
	>30	111	28.60%

**Figure 1:**
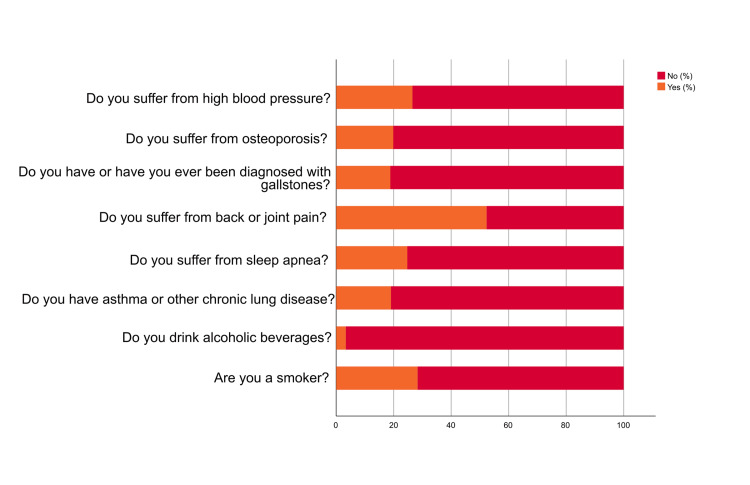
Participants' medical history

**Figure 2:**
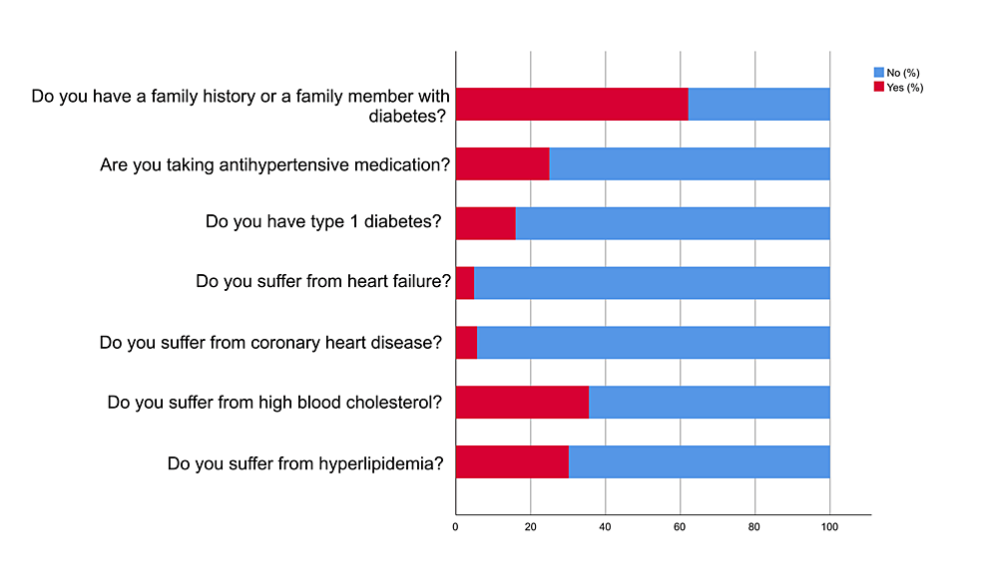
Participants' medical history

Figures 1-2 indicate that the majority of participants were non-smokers, with 71.60% reporting not smoking. Similarly, a significant proportion of participants (96.60%) reported not consuming alcoholic beverages. Regarding chronic lung disease, the majority (80.90%) did not suffer from any chronic lung disease. In terms of sleep apnea, 75.30% did not have sleep apnea. Participants' experiences with back or joint pain were also analyzed. The results showed that 52.30% of participants suffered from back or joint pain. Concerning gallstones, the majority (81.2%) did not have this condition. Regarding osteoporosis, more than half of the participants (80.20%) did not have osteoporosis. Furthermore, 73.50% did not suffer from high blood pressure. Additionally, 69.80% of the participants did not have hyperlipidemia. Similarly, 64.40% of the participants did not have high blood cholesterol. The majority of people (94.30%) did not have coronary heart disease. Additionally, a total of 95.10% did not suffer from heart failure. Regarding type 1 diabetes and the use of antihypertensive medication, a major proportion (84.0%) did not have type 1 diabetes. Moreover, 75.0% did not use antihypertensive medications. Regarding family history of diabetes, the study found that 62.10% of participants had a family history or a family member with diabetes.

Table 3 indicates that the participants identified eating habits as the most perceived cause of obesity, with 40.70% attributing it to this factor. Regarding the perception of obesity as a disease, most participants (85.80%) acknowledged obesity as a disease. Participants' understanding of obesity as an increase in body fat was quite accurate, with 87.40% responding correctly. Regarding exposure to educational messages about the relationship between obesity and diabetes, a significant portion of participants (60.60%) reported having received such messages via e-mail, SMS messages, or social networking sites. When asked if drinking water reduces body weight, most participants (60.60%) correctly believed that it does. The complications of obesity that the participants experienced were illustrated. The most reported complication was type 2 diabetes, with 44.30% of participants indicating its presence. Participants' perceptions of obesity-related risks and factors were also assessed. A majority (86.10%) acknowledged that physical inactivity and excessive sleep are risks for obesity. Regarding genetic factors as a cause of obesity, 69.10% of participants believed it to be true. A significant number of participants (57.70%) reported having an obese family member. Additionally, the majority of participants (86.10%) believed that unhealthy lifestyle habits can cause obesity. Regarding the impact of physical activities on obesity risk, 87.40% of participants correctly believed that physical activities reduce the risk of obesity. When asked about the effect of obesity on insulin sensitivity, the major proportion (67.30%) of participants correctly believed that obesity affects insulin sensitivity. Furthermore, most participants (80.90%) recognized the relationship between obesity, diabetes, and high blood pressure. Lastly, regarding the impact of weight reduction on diabetes control and complications, 82.00% of participants acknowledged that reducing excess weight helps control diabetes and reduces its complications.

**Table 3:** Knowledge about obesity

Parameter	Category	N	%
What do you think are the causes of obesity?	Eating habits	158	40.70%
	Genetic reasons	110	28.40%
	Lack of physical activity	80	20.60%
	Pregnancy	13	3.40%
	Medicines and diseases	26	6.70%
	All	1	0.30%
Obesity is a disease.	No	34	8.80%
	Yes	333	85.80%
	I do not know	21	5.40%
Obesity is an increase in body fat.	No	30	7.70%
	Yes	339	87.40%
	I do not know	19	4.90%
Have you ever seen an educational message about the relationship between obesity to diabetes via e-mail, SMS messages, or social networking sites?	No	116	29.90%
	Yes	235	60.60%
	I do not know	37	9.50%
Does drinking water reduce body weight?	No	58	14.90%
	Yes	235	60.60%
	I do not know	95	24.50%
Do you suffer from any of the following complications of obesity? (more than one answer can be chosen)	No complications	139	35.80%
	Arthritis	112	28.90%
	Difficulty or shortness of breath	59	15.20%
	Cardiovascular disease	46	11.90%
	Type 2 diabetes	172	44.30%
	Gastrointestinal problems (gastric reflux - cholecystitis)	63	16.20%
	High blood pressure and abnormal cholesterol levels	86	22.20%
	Non-alcoholic fatty liver disease	22	5.70%
Physical inactivity and excessive sleep are risks for obesity.	No	19	4.90%
	Yes	334	86.10%
	I do not know	35	9.00%
Obesity is caused by genetic factors.	No	63	16.20%
	Yes	268	69.10%
	I do not know	57	14.70%
A family member living with obesity.	No	139	35.80%
	Yes	224	57.70%
	I do not know	25	6.40%
Unhealthy lifestyle habits can cause obesity.	No	26	6.70%
	Yes	334	86.10%
	I do not know	28	7.20%
Do physical activities reduce obesity risk?	No	25	6.40%
	Yes	339	87.40%
	I do not know	24	6.20%
Does obesity affect the level of insulin sensitivity?	No	29	7.50%
	Yes	261	67.30%
	I do not know	98	25.30%
Obesity causes diabetes and high blood pressure	No	33	8.50%
	Yes	314	80.90%
	I do not know	41	10.60%
Reducing excess weight helps control diabetes and reduces its complications.	No	31	8.00%
	Yes	318	82.00%
	I do not know	39	10.10%

**Figure 3:**
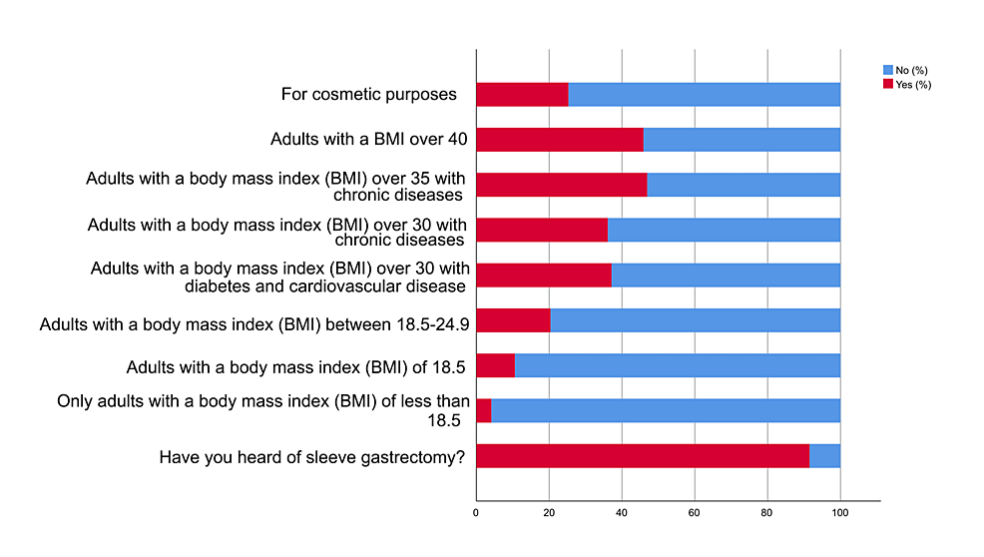
Knowledge about the indication of bariatric surgery

Figure 3 reveals that the majority of participants (91.50%) had heard of sleeve gastrectomy, indicating a relatively high level of awareness about this weight loss surgery. Regarding eligibility criteria based on BMI ranges, most participants (95.90%) were aware of the fact that sleeve gastrectomy is not limited to adults with a BMI of less than 18.5. Similarly, participants demonstrated awareness of sleeve gastrectomy eligibility for adults with a BMI of 18.5. The majority (89.40%) recognized that a BMI of 18.5 is not a reason for sleeve gastrectomy. The understanding of sleeve gastrectomy eligibility for adults with a BMI between 18.5 and 24.9 was also relatively accurate, with 79.60% of participants mistakenly believing that individuals with a BMI between 18.5 and 24.9 were not eligible for sleeve gastrectomy. Participants also exhibited awareness of sleeve gastrectomy eligibility for adults with a BMI over 30 who had diabetes and cardiovascular disease, as well as those with chronic diseases. The majority (62.90%) recognized that individuals with a BMI over 30 with diabetes and cardiovascular disease were eligible for the procedure. Similarly, participants acknowledged that adults with a BMI over 30 with chronic diseases were eligible for sleeve gastrectomy, with 63.90% believing that they were not eligible for gastric sleeve. Regarding eligibility for adults with a BMI over 35 with chronic diseases, the majority (53.10%) believed that adults with a BMI over 35 with chronic diseases were not eligible for the surgery. Participants were asked about the eligibility of adults with a BMI over 40 for sleeve gastrectomy. The largest proportion (54.10%) believed individuals with a BMI over 40 were not eligible for the procedure. Interestingly, when asked about the eligibility of sleeve gastrectomy for cosmetic purposes, 74.70% of participants correctly stated that it was not performed for cosmetic reasons.

**Figure 4:**
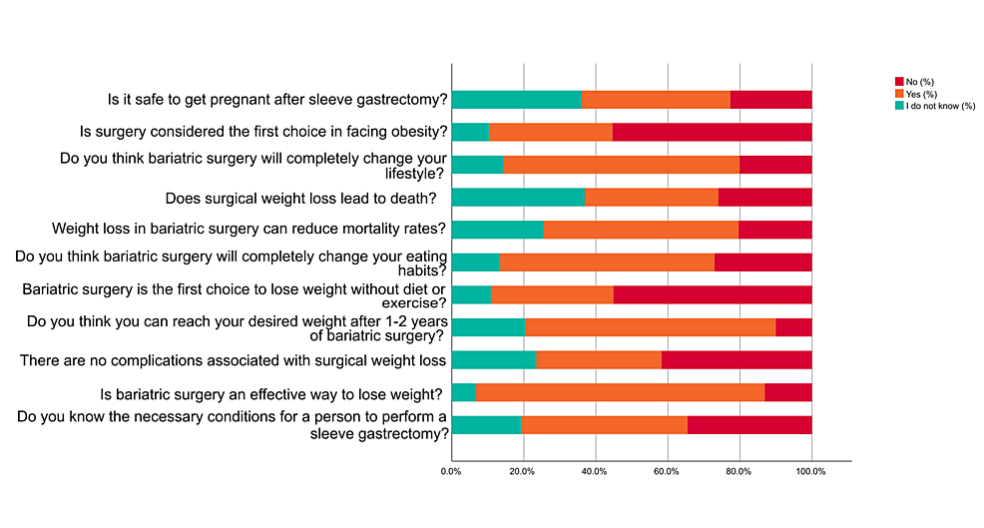
Necessary conditions for a person to undergo bariatric surgery

Based on Figure 4, a significant proportion of participants (46.10%) were aware of the necessary conditions for a person to undergo a sleeve gastrectomy. Regarding the effectiveness of bariatric surgery as a weight loss method, most participants (80.20%) believed that it is an effective way to lose weight. Participants' perceptions about complications associated with surgical weight loss varied, 41.80% of the participants disagreed that there are no associated complications with surgical weight loss. Regarding the potential for reaching the desired weight after one to two years of bariatric surgery, 69.60% of participants believed it was achievable. Participants were also asked whether they considered bariatric surgery the first choice for weight loss without diet or exercise. The majority (55.20%) did not view it as the first choice. Regarding the impact of bariatric surgery on eating habits, the largest percentage (59.50%) of participants believed it would completely change their eating habits. When asked about the potential reduction in mortality rates through weight loss achieved via bariatric surgery, 54.10% of participants believed it could lead to reduced mortality rates. Regarding the perceived risk of death associated with surgical weight loss, opinions were divided. The majority (37.10%) believed it leads to death. Participants' perceptions of bariatric surgery's impact on lifestyle were mixed, with 65.70% believing that it would completely change their lifestyle. Furthermore, 55.40% of the individuals did not view the surgery as the first choice to face obesity. Lastly, regarding the safety of getting pregnant after sleeve gastrectomy, 41.20% of participants believed it was safe.

As shown in Table 4, more than half of the participants (70.10%) were aware of the complications of gastric sleeve. Regarding the severe complications of gastric sleeve, internal bleeding is the most common complication (41.00%), while anemia is the most common chronic complication of gastric sleeve (35.10%). Most participants (62.40%) did not have any family members having sleeve gastrectomy. Only 57.50% had one of their family members having sleeve gastrectomy. Regarding the surgical technique used, 84.90% of the participants had gastric sleeve as the surgical technique. Most of the participants (30.10%) disagreed that it is possible to stop exercising and eat all kinds of nutritional foods after reaching the ideal weight.

**Table 4:** Severe and chronic complications of sleeve gastrectomy

Parameter	Category	N	%
Have you heard of complications of gastric sleeve?	No	116	29.90%
	Yes	272	70.10%
If your answer is yes to the previous question, what are the severe complications of sleeve gastrectomy?	I do not know	105	27.10%
	Internal bleeding	159	41.00%
	Anemia	134	34.50%
	Suppuration (pus collection) after surgery	96	24.70%
	Wrapping the stomach around itself	40	10.30%
	Iron deficiency	104	26.80%
	Stomach content leakage	93	24.00%
	Neuropathies	45	11.60%
	Pulmonary vascular occlusion	29	7.50%
	Lack of mineral salts in the body	76	19.60%
	Overweight	54	13.90%
What are the chronic complications of gastric sleeve surgery? (you can choose more than one option)	I do not know	117	30.20%
	Internal bleeding	112	28.90%
	Anemia	136	35.10%
	Suppuration (pus collection) after surgery	56	14.40%
	Wrapping the stomach around itself	51	13.10%
	Iron deficiency	135	34.80%
	Stomach content leakage	59	15.20%
	Neuropathies	58	14.90%
	Pulmonary vascular occlusion	37	9.50%
	Lack of mineral salts in the body	97	25.00%
	Overweight	62	16.00%
Have you or any of your family members ever had a sleeve gastrectomy?	No	242	62.40%
	Yes	146	37.60%
Who did sleeve gastrectomy?	Me	56	38.40%
	One of the family members	84	57.50%
	Me and some of family members	1	0.70%
	My friend/s	4	2.70%
	No one	1	0.70%
What is the surgical technique that you did?	Gastric sleeve	124	84.90%
	Gastric banding	8	5.50%
	Gastric balloon	11	7.50%
	Gastric bypass	3	2.10%
Is it possible to stop exercising and eat all kinds of nutritional foods after reaching the ideal weight?	Strongly disagree	25	17.10%
	Disagree	44	30.10%
	Neutral	42	28.80%
	Agree	21	14.40%
	Strongly agree	14	9.60%

**Figure 5:**
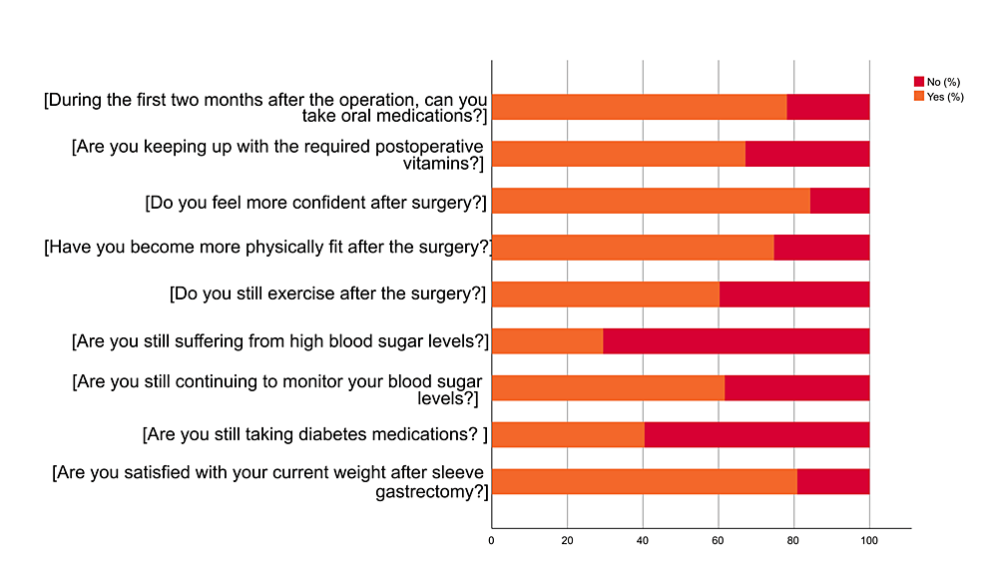
Adherence of the participants after sleeve gastrostomy

**Figure 6:**
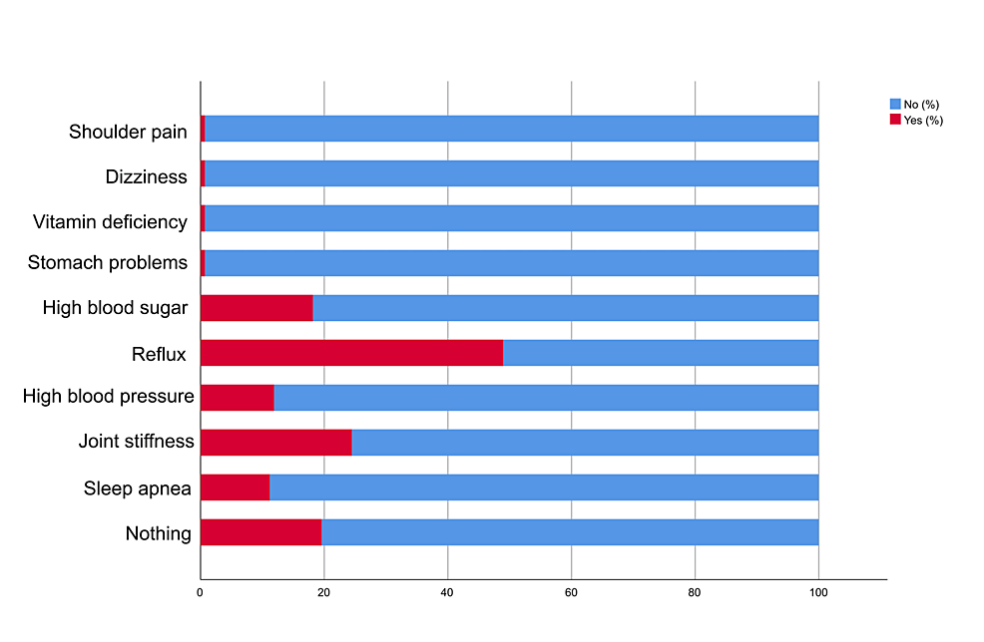
Chronic conditions after sleeve gastrectomy

Around (80.80%) of the participants were satisfied with the current weight after sleeve gastrectomy. Regarding diabetes medications, the largest proportion (59.60%) stopped taking diabetes medications. In addition, the majority of individuals (61.60%) were still continuing to monitor blood sugar levels, while the majority (70.50%) achieved remission. Regarding exercising after the surgery, the largest percentage (60.30%) was still exercising after the surgery. Furthermore, 74.70% of the participants became more physically fit after the surgery and (84.20%) of them felt more confident after the surgery. Additionally, the majority proportion (67.10%) of the participants were keeping up with the required post-operative vitamins. Based on the ability to take oral medications during the first two months after the operation, 78.10% of the individuals could take oral medications (Figure 5). In Figure 6, the majority proportions of the participants reported remission of sleep apnea (88.80%), joint stiffness (75.50%), high blood pressure (88.10%), reflux (51.00%), high blood sugar (81.80%), stomach problems (99.30%), vitamin deficiency (99.30%), dizziness (99.30%), and shoulder pain (99.30%) after the surgery.

Based on Table 5, regarding gender, the majority of females 206 (94.10%) and males 149 (88.20%) have heard of sleeve gastrectomy, and the difference was statistically significant (P-value = 0.039). Furthermore, the majority of Saudi (317, 92.70%) and non-Saudi (38, 82.60%) participants have heard of sleeve gastrectomy (P-value = 0.021). There was no significant difference in awareness about sleeve gastrectomy based on age, marital status, residential area, educational level, monthly income, and BMI. Based on the multivariate regression analysis (Table 6), non-Saudi participants were predicted to have lower awareness about bariatric surgery compared to Saudi participants (OR = 0.403, 95% CI (0.168-0.965), P-value = 0.041).

**Table 5:** Factors associated with awareness about bariatric surgery

Parameter	Category	Have you heard of sleeve gastrectomy?				P value
		No		Yes		
		N	%	N	%	
Age	18-23	9	10.30%	78	89.70%	0.186
	24-30	11	14.30%	66	85.70%	
	31-40	4	5.60%	67	94.40%	
	41-50	4	5.90%	64	94.10%	
	51-60	2	3.30%	58	96.70%	
	>60	3	12.00%	22	88.00%	
Gender	Male	20	11.80%	149	88.20%	0.039
	Female	13	5.90%	206	94.10%	
Marital status	Single	15	9.90%	136	90.10%	0.495
	Married	13	6.60%	183	93.40%	
	Divorced	2	9.50%	19	90.50%	
	Widowed	3	15.00%	17	85.00%	
Nationality	Saudi	25	7.30%	317	92.70%	0.021
	Non-Saudi	8	17.40%	38	82.60%	
Residential area	From the governorates of Makkah	27	8.80%	279	91.20%	0.664
	Outside the governorates of Makkah	6	7.30%	76	92.70%	
Educational level	High school education or less	13	12.00%	95	88.00%	0.234
	Bachelor's	19	7.50%	233	92.50%	
	High education	1	3.60%	27	96.40%	
Monthly income	<5,000	14	10.40%	120	89.60%	0.883
	5,000-10,000	8	8.20%	90	91.80%	
	10,000-15,000	6	6.50%	86	93.50%	
	15,000-20,000	3	7.90%	35	92.10%	
	>20,000	2	7.70%	24	92.30%	
BMI	Underweight (18.49 or less)	0	0.00%	8	100.00%	0.892
	Normal weight (18.5-24.9)	6	7.80%	71	92.20%	
	Overweight (25-29.9)	10	11.00%	81	89.00%	
	Class I obesity (30-34.9)	5	7.20%	64	92.80%	
	Class II obesity (35-39.9)	5	8.60%	53	91.40%	
	Class III obesity (40 or more)	6	8.10%	68	91.90%	

**Table 6:** Predictors of awareness about bariatric surgery

Parameter	Category	OR	95% CI		P value
			LB	UB	
Gender	Male	Ref.	Ref.	Ref.	Ref.
	Female	2.017	0.967	4.209	0.061
Nationality	Saudi	Ref.	Ref.	Ref.	Ref.
	Non-Saudi	0.403	0.168	0.965	0.041

## Discussion

Metabolic bariatric surgeries (MBS) have recently become a trend for people living with obesity with or without associated medical problems. Obesity is a major risk factor for diabetes T2DM. MBS can lead to marked improvements in glucose tolerance and insulin sensitivity in obese patients. This study aimed to assess awareness of the benefits of bariatric surgery among patients with diabetes in the Makkah region of Saudi Arabia.

The study surveyed 388 individuals older than 18, over half of whom 58.00% reported having been diagnosed with type 2 diabetes [24], which indicates a high prevalence of diabetes.

The present study found high awareness of obesity as a disease (85.8%), and 80.90% were aware that obesity is associated with many metabolic complications, T2DM, and hypertension. These findings were also seen in another study by Twig et al. [25]. Additionally, 67.3% of participants correctly identified the effect of obesity on insulin sensitivity, consistent with a 2022 study by Alharbi et al. [26] of 421 T2DM, 55.6% of whom were living with obesity.

However, awareness of bariatric surgery was high among the participants, with 91.5% having heard of the surgery. The high level of awareness about sleeve gastrectomy surgery observed in this study corresponds with previous research conducted in Saudi Arabia by Alzahrani et al. [24]. This indicates sleeve gastrectomy has become a well-known bariatric procedure among the Saudi population in recent years.

Based on the results of this study, most participants believe that bariatric surgery is the most effective means of weight reduction, but only 46.10% demonstrated awareness of the required eligibility criteria for a bariatric surgery procedure. The observed level of awareness is relatively lower than previously reported findings in the Al Qassim region of Saudi Arabia in 2021 by Alolayan et al. [27], who found that 60.90% were knowledgeable about indicators for sleeve gastrectomy surgery. Therefore, although most of the participants believed bariatric surgery may treat chronic disease problems and reduce mortality, they did not consider bariatric surgery as the first choice for weight loss without diet or exercise. This seems to indicate that non-invasive weight loss strategies are preferred to surgical procedures. Moreover, a large proportion of participants expected substantial changes in their dietary habits and lifestyles after surgery. This emphasizes the need for extensive prior and postoperative counseling to help patients understand these changes as well as the role of a multidisciplinary approach in the management.

Regarding complications, the current study found that high awareness (70.1%) of bariatric surgery internal bleeding (41%) and anemia (35.1%) were most commonly identified; this was also seen in Alayaaf et al. [28].

Additionally, this study measured the effectiveness of bariatric surgery among 37.6% of participants who underwent bariatric surgery. This study’s findings indicate positive outcomes and high patient satisfaction following sleeve gastrectomy. The majority of patients reported satisfaction with their weight loss, discontinuation of diabetes medications, and improved blood sugar control. Furthermore, patients demonstrated adherence to postoperative vitamin supplementation and continued engagement in exercise, leading to enhanced physical fitness and confidence levels. The significant reduction in postoperative symptoms shows that sleeve gastrectomy improves overall health. The study also found that sex and nationality were significant predictors of awareness. Females and Saudi nationals were more likely to have heard of sleeve gastrectomy. However, awareness levels did not differ based on other sociodemographic factors, such as age, education, or income. This could indicate the need for targeted health communication strategies to enhance awareness and understanding across diverse demographic groups.

The benefits of sleeve gastrectomy for diabetic patients can be promoted to those in need through targeted educational initiatives. 

Bariatric surgery should be used if weight loss and the treatment of co-morbidities related to obesity conditions that raise mortality and lower quality of life are demonstrated [28].

Limitations

Regardless of the data from this study, there are some limitations. The cross-sectional design of this study limits the ability to draw conclusions. The weight and height of participants were self-reported. Additionally, the data were collected randomly in the Makkah region of Saudi Arabia, so the study results will not reflect the awareness of the general population of Saudi Arabia. Only antidiabetic medications were measured in this study; other medications associated with weight reduction were not measured.

Finally, the variables investigated failed to adjust for all the various demographic, socioeconomic, and health-related factors that could affect people's awareness of bariatric surgery. These preliminary findings on knowledge of bariatric surgery and related factors require further study with larger, more representative groups to be confirmed and expanded upon.

## Conclusions

This study examined awareness about the benefits of bariatric surgery for T2DM among 388 Saudi and non-Saudi diabetic patients. Most participants showed a high awareness of obesity and its risk to health; additionally, more than 91% had heard about bariatric surgery. However, there was a lower level of knowledge about eligibility criteria, expected outcomes, and lifestyle changes required after bariatric surgery. This study provides insights into knowledge of bariatric surgery and its outcomes among Saudi patients that are useful for improving care delivery and access. More research can be done to verify the long-term sustainability of weight loss and diabetes remission after surgery.

Overall, this study provides insights into knowledge of bariatric surgery and its outcomes among patients with diabetes that are useful for improving healthcare access in the future.
